# A non-recurrent inferior laryngeal nerve in a man undergoing thyroidectomy: a case report

**DOI:** 10.1186/1752-1947-4-386

**Published:** 2010-11-29

**Authors:** Diogo Casal, António Peças, Daniel Sousa, Jorge Rosa-Santos

**Affiliations:** 1 Head and Neck Surgery Department, Instituto Português de Oncologia de Lisboa, R. Prof. Lima Basto, 1099-023 Lisbon, Portugal

## Abstract

**Introduction:**

A non-recurrent variant of the inferior laryngeal nerve has been seldom reported. These reports are mostly based on cadaveric dissection studies or large chart review studies in which the emphasis is placed on the determination of the frequency of the variation, and not on the clinical appearance of this variant. We graphically describe the intraoperative identification of a non-recurrent inferior laryngeal nerve.

**Case Presentation:**

A 44-year old Caucasian man was referred to the Head and Neck Surgery Outpatient Clinic with the diagnosis of a nodular mass in his left thyroid lobe that had been growing for one year. A fine needle aspiration puncture was compatible with thyroid papillary cancer. It was decided that the patient should undergo total thyroidectomy. During surgery, a non-recurrent right inferior laryngeal nerve was noted. This nerve emanated from the right vagus nerve, entering the larynx 3 cm after its origin. The nerve did not show a recurrent course. The nerve on the left side had a normal configuration. The surgery and post-operative period were uneventful, and the patient had no change in his voice.

**Conclusion:**

This paper allows those interested to become acquainted with the normal intraoperative appearance of a non-recurrent inferior laryngeal nerve. This will undoubtedly be of significance for all of those performing invasive diagnostic and surgical procedures in the neck and upper thoracic regions, in order to minimize the risk of iatrogenic injury to this nerve. This is of extreme importance, since a unilateral lesion of this nerve may result in permanent hoarseness, and a bilateral lesion may lead to aphonia and life-threatening dyspnea.

## Introduction

The inferior laryngeal nerve (ILN) is traditionally named recurrent due to the fact that, branching off the vagus nerve, it usually describes a loop as it turns upwards, passing under the subclavian artery on the right and under the *ligamentum arteriosum *on the left [1].

The ILN provides innervation to all larynx intrinsic muscles, except for the cricothyroid muscle. From a sensorial point of view, it innervates the mucosal surface of the larynx below the vocal cords [2]. Injury to this nerve may thus result in paralysis of the vocal cord on the same side, leading to permanent hoarseness. If the lesion is bilateral, aphonia and life-threatening dyspnea may ensue as a result of medial placement of the paralytic vocal cords, which can obstruct the glottis [3].

The relatively long course of the inferior laryngeal nerves place them at risk of iatrogenic injury in numerous procedures involving the cervical and upper thoracic regions [1]. Among these, surgical procedures in the cervical region, namely thyroidectomies, are particularly common. In this last setting, for example, permanent injury to the recurrent laryngeal nerve (ILN) is reported in 0.25 to 2.6% of cases, with rates >8% in case of reoperation [4]. It has been demonstrated that dissection and visualization of the ILN during such procedures significantly reduces the risk of lesion to this nerve [5]. To accomplish this, it is instrumental to have a sound knowledge of the normal and variant forms of the ILN [6].

## Case presentation

A 44-year-old Caucasian man was referred to the Head and Neck Surgery Outpatient Clinic with the diagnosis of a nodular mass in his left thyroid lobe that had been growing since the previous year. His previous medical history was unremarkable. No other enlarged masses or lymph nodes were palpated in the physical examination or detected in the imagiological study of the neck with ultrasound and computed tomography (CT) scan. A fine needle aspiration puncture was performed and the result was compatible with thyroid papillary cancer. In the Group Decision Clinic it was decided that the patient should undergo total thyroidectomy.

**Figure 1:**
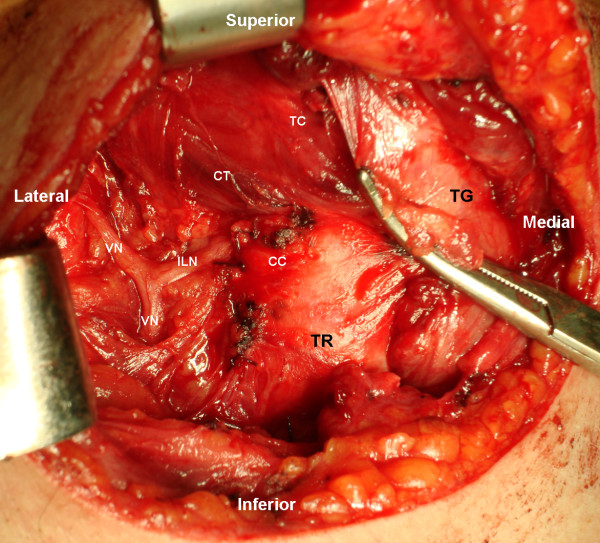
**Intra-operative right lateral view of the neck**. The right Inferior Laryngeal Nerve (ILN) is seen originating perpendicularly from the Vagus Nerve (VN) and entering the Larynx between the Crycoid Cartilage (CC) and the Thyroid Cartilage (TC), just below the Crico-Thyroid Muscle (CT). The Thyroid Gland (TG) is being retracted medially to increase exposure of ILN and allowing visualization of the Trachea (TR). The right ILN does not have any recurrent path.

During surgery, a non-recurrent right inferior laryngeal nerve was noted (Figure 1). This nerve emanated from the right vagus nerve almost at a straight angle, entering the larynx 3 cm after its origin. The nerve did not show a recurrent course. The nerve on the left side had a normal configuration coming upwards in the tracheo-esophageal groove, in a recurrent fashion, presumably from the left vagus nerve. The surgery and post-operative period was uneventful, and our patient had no change in his voice.

## Discussion

The reported incidence of the non-recurrent ILN is widely variable. In some series, the proportion of this variation is null [3-7] whereas in others it is as high as 3.9% [8-10]. In the largest series reported, including 6637 observations of the ILN during neck surgery, the frequency of the non-recurrent ILN was 0.54% (17 cases in 3098) on the right and 0.07% on the left (2 cases in 2846), corresponding to a global prevalence of 0.32% [11].

Despite the discrepancies in the relative proportion of this variant of the ILN, most authors agree that this variant is most common on the right side. The embryological basis for this finding seems to be a vascular disorder known as *arteria lusoria *in which the fourth right aortic arch is abnormally absorbed [12]. Consequently, this vessel fails to drag the right recurrent laryngeal nerve (ILN) caudally when the heart descends, and the neck elongates during embryonic development [9,13,14]. This anomaly generally leads to a right subclavian artery that originates as a branch of a normal aortic arch and passes upward to the right behind the esophagus. The incidence of this vascular malformation is reportedly as high as 0.5 to 2% of the general population [15]. Even though it is usually asymptomatic, nearly 5% of these patients experience dysphagia (*dysphagia lusoria*) or symptoms related to artery tortuosity, and premature atherosclerosis. Rarely, it is associated with aneurism formation [15].

On the left side, a non-recurrent ILN has only been observed in cases of dextrocardia [13].

Theoretically, the pre-operative diagnosis of a non-recurrent ILN could be attempted with imaging studies, namely CT scan or magnetic resonance imaging (MRI), to visualize the *arteria lusoria *[14]. However, although the presence of anatomical variants of the ILN have been associated with a higher risk of iatrogenic injury during surgery in the head and upper thorax region [4], it has not yet been shown that the systematic use of pre-operative imaging studies can minimize this risk.

## Conclusion

Invasive procedures in the head and neck region that may compromise the ILN are part of everyday clinical and surgical practice. A sound knowledge of the normal morphology and most frequent variants of the ILN, including its non-recurrent variant, can help doctors to minimize the risk of iatrogenic lesion to this nerve.

## Consent

Written informed consent was obtained from the patient for publication of this case report and accompanying images. A copy of the written consent is available for review by the Editor-in-Chief of this journal

## Competing interests

The authors declare that they have no competing interests.

## Authors' contributions

All authors have read and approved the final manuscript. DC played a major role in writing the manuscript and analyzed the patient's data. AP aided in the editing of the manuscript and analyzed the patient's data. DS participated in the writing and editing of the manuscript and analyzed the patient's data. JRS aided in the editing of the manuscript and analyzed the patient's data.

## References

[B1] Khaki AA Tubbs RS Shoja MM Zarrintan S An unusual course of the left recurrent laryngeal nerve Clin Anat 2007 20 344 346 10.1002/ca.20341 17031863

[B2] Partab P Hurrinarain K Ramsaroop L Satyapal KS Atypical anastomosis of laryngeal nerves Clin Anat 2006 19 651 656 10.1002/ca.20306 16583419

[B3] Lee MS Lee UY Lee JH Han SH Relative direction and position of recurrent laryngeal nerve for anatomical configuration Surg Radiol Anat 2009 31 649 655 10.1007/s00276-009-0494-y 19326038

[B4] Casella C Pata G Nascimbeni R Mittempergher F Salerni B Does extralaryngeal branching have an impact on the rate of postoperative transient or permanent recurrent laryngeal nerve palsy? World J Surg 2009 33 261 265 10.1007/s00268-008-9832-1 19023612

[B5] Hermann M Alk G Roka R Glaser K Freissmuth M Laryngeal recurrent nerve injury in surgery for benign thyroid diseases: effect of nerve dissection and impact of individual surgeon in more than 27,000 nerves at risk Ann Surg 2002 235 261 268 10.1097/00000658-200202000-00015 11807367 PMC1422423

[B6] Makay O Icoz G Yilmaz M Akyildiz M Yetkin E The recurrent laryngeal nerve and the inferior thyroid artery--anatomical variations during surgery Langenbecks Arch Surg 2008 393 681 685 10.1007/s00423-008-0320-8 18330594

[B7] Arantes AGS Rubinstein F Oliveira R Anatomia microcirúrgica do nervo laríngeo recorrente: aplicações no acesso cirúrgico anterior à coluna cervical Arq Neuro Psiquiatr 2004 62 5 10.1590/s0004-282x2004000400026 15334235

[B8] Page C Monet P Peltier J Bonnaire B Strunski V Non-recurrent laryngeal nerve related to thyroid surgery: report of three cases J Laryngol Otol 2008 122 757 761 10.1017/S0022215107008389 17517167

[B9] Nakatani T Tanaka S Mizukami S Anomalous triad of a left-sided inferior vena cava, a retroesophageal right subclavian artery, and bilateral superficial brachial arteries in one individual Clin Anat 1998 11 112 117 10.1002/(SICI)1098-2353(1998)11:2<112::AID-CA8>3.0.CO;2-W 9509924

[B10] Abboud B Aouad R Non-recurrent inferior laryngeal nerve in thyroid surgery: report of three cases and review of the literature J Laryngol Otol 2004 118 139 142 10.1258/002221504772784603 14979952

[B11] Henry JF Audiffrit J Plan M The nonrecurent inferior laryngeal nerve. A propos of 19 cases including 2 on the left side J Chir (Paris) 1985 122 7 4044700

[B12] Avisse C Marcus C Delattre JF Cailliez-Tomasi JP Palot JP Ladam-Marcus V Menanteau B Flament JB Right nonrecurrent inferior laryngeal nerve and arteria lusoria: the diagnostic and therapeutic implications of an anatomic anomaly. Review of 17 cases Surg Radiol Anat 1998 20 227 232 9706684

[B13] Defechereux T Albert V Alexandre J Bonnet P Hamoir E Meurisse M The inferior non recurrent laryngeal nerve: a major surgical risk during thyroidectomy Acta Chir Belg 2000 100 62 67 10925715

[B14] Schneider J Baier R Dinges C Unger F Retroesophageal right subclavian artery (lusoria) as origin of traumatic aortic rupture Eur J Cardiothorac Surg 2007 32 385 387 10.1016/j.ejcts.2007.04.034 17560115

[B15] Attmann T Brandt M Muller-Hulsbeck S Cremer J Two-stage surgical and endovascular treatment of an aneurysmal aberrant right subclavian (Lusoria) artery Eur J Cardiothorac Surg 2005 27 1125 1127 10.1016/j.ejcts.2005.02.029 15896634

